# Serologic extracellular matrix remodeling markers are related to fibrosis stage and prognosis in a phase 2b trial of simtuzumab in patients with primary sclerosing cholangitis

**DOI:** 10.1097/HC9.0000000000000467

**Published:** 2024-07-05

**Authors:** Douglas Thorburn, Diana J. Leeming, William T. Barchuk, Ya Wang, Xiaomin Lu, Vladislav A. Malkov, Kaori L. Ito, Christopher L. Bowlus, Cynthia Levy, Zachary Goodman, Morten A. Karsdal, Andrew J. Muir, Jun Xu

**Affiliations:** 1 Sheila Sherlock Liver Centre and UCL Institute for Liver and Digestive Health, Royal Free Hospital, London, UK; 2 Nordic Bioscience A/S, Herlev, Denmark; 3 Gilead Sciences, Inc., Foster City, California, USA; 4 Division of Gastroenterology and Hepatology, University of California Davis School of Medicine, Sacramento, California, USA; 5 Division of Digestive Health and Liver Diseases, Miller School of Medicine, University of Miami, Miami, Florida, USA; 6 Inova Fairfax Hospital, Falls Church, Virginia, USA; 7 Duke Clinical Research Institute, Duke University School of Medicine, Durham, North Carolina, USA

## Abstract

**Background:**

Novel noninvasive predictors of disease severity and prognosis in primary sclerosing cholangitis (PSC) are needed. This study evaluated the ability of extracellular matrix remodeling markers to diagnose fibrosis stage and predict PSC-related fibrosis progression and clinical events.

**Methods:**

Liver histology and serum markers of collagen formation (propeptide of type III collagen [Pro-C3], propeptide of type IV collagen, propeptide of type V collagen), collagen degradation (type III collagen matrix metalloproteinase degradation product and type IV collagen matrix metalloproteinase degradation product), and fibrosis (enhanced liver fibrosis [ELF] score and its components [metalloproteinase-1, type III procollagen, hyaluronic acid]) were assessed in samples from baseline to week 96 in patients with PSC enrolled in a study evaluating simtuzumab (NCT01672853). Diagnostic performance for advanced fibrosis (Ishak stages 3–6) and cirrhosis (Ishak stages 5–6) was evaluated by logistic regression and AUROC. Prognostic performance for PSC-related clinical events and fibrosis progression was assessed by AUROC and Wilcoxon rank-sum test.

**Results:**

Among 234 patients, 51% had advanced fibrosis and 11% had cirrhosis at baseline. Baseline Pro-C3 and ELF score and its components provided moderate diagnostic ability for discrimination of advanced fibrosis (AUROC 0.73–0.78) and cirrhosis (AUROC 0.73–0.81). Baseline Pro-C3, ELF score, and type III procollagen provided a moderate prognosis for PSC-related clinical events (AUROC 0.70–0.71). Among patients without cirrhosis at baseline, median changes in Pro-C3 and ELF score to week 96 were higher in those with than without progression to cirrhosis (both *p* < 0.001).

**Conclusions:**

Pro-C3 correlated with fibrosis stage, and Pro-C3 and ELF score provided discrimination of advanced fibrosis and cirrhosis and predicted PSC-related events and fibrosis progression. The results support the clinical utility of Pro-C3 and ELF score for staging and as prognostic markers in PSC.

## Graphical abstract

**Figure:**
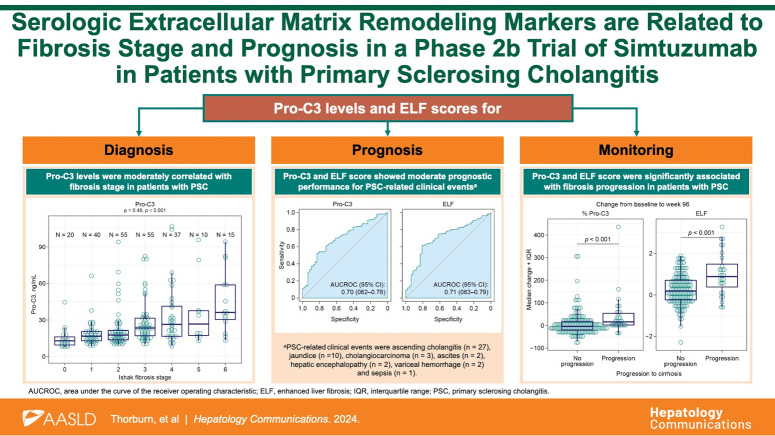

## IMPACT AND IMPLICATIONS

Primary sclerosing cholangitis (PSC) is an inflammatory liver disease with progressive fibrosis and eventually cirrhosis. Here, we show that serum levels of the collagen fragment and fibrosis markers Pro-C3 and ELF score relate to fibrosis stage and can predict PSC-related clinical events and monitor fibrosis progression. Our results support the clinical use of Pro-C3 and ELF score as noninvasive markers in PSC.

## INTRODUCTION

Primary sclerosing cholangitis (PSC) is a chronic cholestatic liver disease characterized by inflammation and fibrosis of the bile ducts.^1^ In most patients with PSC, the liver disease progresses slowly, with progressive hepatobiliary fibrosis and eventually cirrhosis.^1^,^2^ Magnetic resonance cholangiopancreatography is used for diagnosis and follow-up in patients with PSC, with liver biopsy performed if PSC is suspected but magnetic resonance cholangiopancreatography is normal.^1^,^3^ Outside of the clinical trial setting, liver biopsy is not recommended for fibrosis staging or as a prognostic predictor in PSC.^1^

There is an unmet need for noninvasive markers in PSC to assess disease activity and prognosis. In particular, there is a need for validated intermediary surrogate biomarkers for histological disease progression and clinical events to help support the prediction of treatment efficacy within early-phase (phase 2) clinical trials. Several markers, including serum extracellular matrix (ECM) fragments, have been assessed as secondary or exploratory endpoints in short-duration phase 2 trials in PSC^4^,^5^,^6^; however, longer-duration trials are needed to demonstrate correlations between levels of these markers and histological and clinical disease progression.

Serum markers of collagen formation, collagen degradation, and fibrosis have the potential to reflect fibrosis stage and prognosis in PSC. Fibrosis progression involves increased turnover of the ECM scaffolding and formation of fibrotic scars, composed principally of collagens.^7^ The ECM is a lattice-like formation, with the interstitial ECM being composed mainly of fibril collagens type I, III, and V, and the basal lamina ECM consisting mostly of collagen type IV.^7^ Dysregulation of ECM remodeling and fibril collagen formation leads to functional and structural alterations in liver fibrosis.

Lysyl oxidase-like protein 2 is an amine oxidase that crosslinks collagen and elastin and promotes stabilization of the ECM.^8^ In a phase 2b study in patients with PSC, the monoclonal humanized anti-lysyl oxidase-like protein 2 antibody simtuzumab failed to show clinical benefit or signs of fibrosis improvement among treatment groups.^2^ The study provided detailed data on the natural history of PSC.^2^ Overall, at baseline, 51% of the 234 patients included had advanced fibrosis or cirrhosis, median hepatic collagen content on liver biopsy staining was 4.4%, alpha-smooth muscle actin (α-SMA) expression was 2.5%, and median enhanced liver fibrosis (ELF) score was 9.46. The objective of the current work was to evaluate correlations of ECM markers with liver fibrosis, their diagnostic performance for advanced fibrosis and cirrhosis, and their prognostic utility for PSC-related clinical events and fibrosis progression.

## METHODS

### Study design and patients

Data were from a 96-week, phase 2b, multicenter, randomized, double-blind, placebo-controlled study of simtuzumab in patients with PSC (NCT01672853). The methods and primary results of the simtuzumab phase 2 study have been described.^2^ Briefly, the study enrolled patients aged 18–70 years who had chronic (persisting for ≥ 6 mo) cholestatic liver disease caused by PSC. Key eligibility criteria included PSC confirmed by liver biopsy and magnetic resonance cholangiopancreatography. The study excluded patients with moderately or severely active ulcerative colitis (defined as partial Mayo score > 4, bleeding subscore > 1, or current use of oral corticosteroids and/or antagonists of TNF-α or α4β7 integrin). Patients were randomized in a 1:1:1 ratio to receive 75 mg simtuzumab, 125 mg simtuzumab, or placebo by subcutaneous injection every week for 96 weeks. The study protocol was approved by the review board or ethics committee of each institution, and the study was conducted in accordance with the International Conference on Harmonization Good Clinical Practice Guidelines and the Declaration of Helsinki. Written informed consent was obtained from each patient included in the study. Study outcomes were similar for the simtuzumab and placebo groups^2^; data from patients in all treatment groups were thus combined for the present analyses.

### Assessments

Liver biopsies were obtained at baseline and weeks 48 and 96, and were assessed centrally, with morphometric quantification of hepatic collagen content and α-SMA expression. Fibrosis was staged according to the Ishak classification.^9^ Ishak stages 3–6 indicate advanced fibrosis and stages 5–6 indicate cirrhosis. Serum samples obtained at baseline and weeks 12, 24, 48, and 96 were used to assess levels of the collagen formation markers propeptide of type III collagen (Pro-C3), propeptide of type IV collagen, and propeptide of type V collagen, the collagen degradation markers C3M and type IV collagen matrix metalloproteinase degradation product (all Nordic Bioscience, Herlev, Denmark), and the ELF score fibrosis markers TIMP-1, type III procollagen (PIII-NP), and hyaluronic acid (ELF test; Siemens Healthcare, Erlangen, Germany).^10^ PSC-related clinical events (ascending cholangitis, jaundice, ascites, sepsis, cholangiocarcinoma, HE, variceal hemorrhage, HCC, and death) were assessed during follow-up.

### Statistical analyses

Analyses were performed using R and SAS 9.4 (SAS Institute). Spearman correlations were used to evaluate baseline associations between ECM markers and liver fibrosis stage and between Pro-C3 and hepatic collagen content, α-SMA expression, and ELF scores. Spearman correlation coefficients (*ρ*) of at least 0.60 were regarded as strong, values of 0.40–0.59 as moderate, and values below 0.40 as weak.^11^ The diagnostic performance of ECM markers for advanced fibrosis (Ishak stages 3–6 vs. 0–2) and cirrhosis (Ishak stages 5–6 vs. 0–4) was evaluated by AUROC from a univariate logistic regression model. AUROC values of at least 0.8 were regarded as strong, values of 0.7–0.8 as moderate, and values below 0.7 as weak.^12^ The Wilcoxon rank-sum test was used to determine if the median marker levels are different in patients with versus without advanced fibrosis and in patients with versus without cirrhosis.

Optimal diagnostic cutoffs of baseline Pro-C3, ELF score, and ELF score components TIMP-1, PIII-NP, and hyaluronic acid, which provide optimal discrimination of advanced fibrosis, were explored using 3 different methods. The High Youden method identifies an optimal cutoff that maximizes the Youden index, which is defined as sensitivity + specificity − 1. The high-sensitivity method identifies an optimal cutoff that maximizes specificity among those with sensitivity ≥0.85. The high-specificity method identifies an optimal cutoff that maximizes sensitivity among those with specificity ≥0.85. The diagnostic performance of the composite markers “age, presence of diabetes, Pro-C3, and platelet count” (ADAPT) and fibulin-3 (FIBC3) for advanced fibrosis was assessed by the AUROC from a univariate logistic regression model; ADAPT and FIBC3 scores at baseline were calculated using published algorithms developed for metabolic dysfunction-associated steatotic liver disease.^13^,^14^ The prognostic performance of baseline ECM markers on PSC-related clinical events was evaluated using the HR from a univariate Cox proportional hazards regression model and AUROC from the univariate logistic regression model.

Prognostic performance of ECM markers on fibrosis progression was evaluated according to the patient’s change in fibrosis stage from baseline to postbaseline visit at week 48 and week 96. Prognostic performance was evaluated in patients with data available at both baseline and at week 48 or week 96. When data were not available at week 96, data were imputed using available week 48 data. The change in fibrosis was classified into one of 3 categories: “fibrosis regression” (when change < 0), “no change in fibrosis” (when change = 0), and “fibrosis progression” (when change > 0). Jonckheere’s trend test was used to determine if there was an increasing or decreasing trend in median change from baseline in marker levels in the ordinal groups of “fibrosis regression,” “no change in fibrosis,” and “fibrosis progression.” The prognostic performance of ECM markers on fibrosis progression was also evaluated in patients without cirrhosis at baseline, among whom the median changes from baseline in marker levels were compared between those who progressed to cirrhosis and those who remained without cirrhosis at postbaseline using the Wilcoxon rank-sum test.

Intraindividual coefficients of variation for Pro-C3, ELF score, PIII-NP, TIMP-1, and hyaluronic acid were assessed using data collected at baseline, week 12, and week 24 in patients with available data at all visits, as well as in the subsets of patients whose baseline Ishak fibrosis stages were 0–2, 3–4, and 5–6. Specifically, a random intercept model was fitted, assuming constant between- and within-patient variation over time. The intraindividual coefficient of variation was calculated as the estimated within-patient SD divided by the estimated mean.

## RESULTS

### Patients

Data were included from all 234 patients who participated in the simtuzumab phase 2 clinical trial. Baseline patient demographic and clinical characteristics are listed in Supplemental Table S1, http://links.lww.com/HC9/A935, as described.^2^ Overall, 51% of patients had advanced fibrosis and 11% had cirrhosis.

### Correlations of ECM markers with liver fibrosis

**FIGURE 1:**
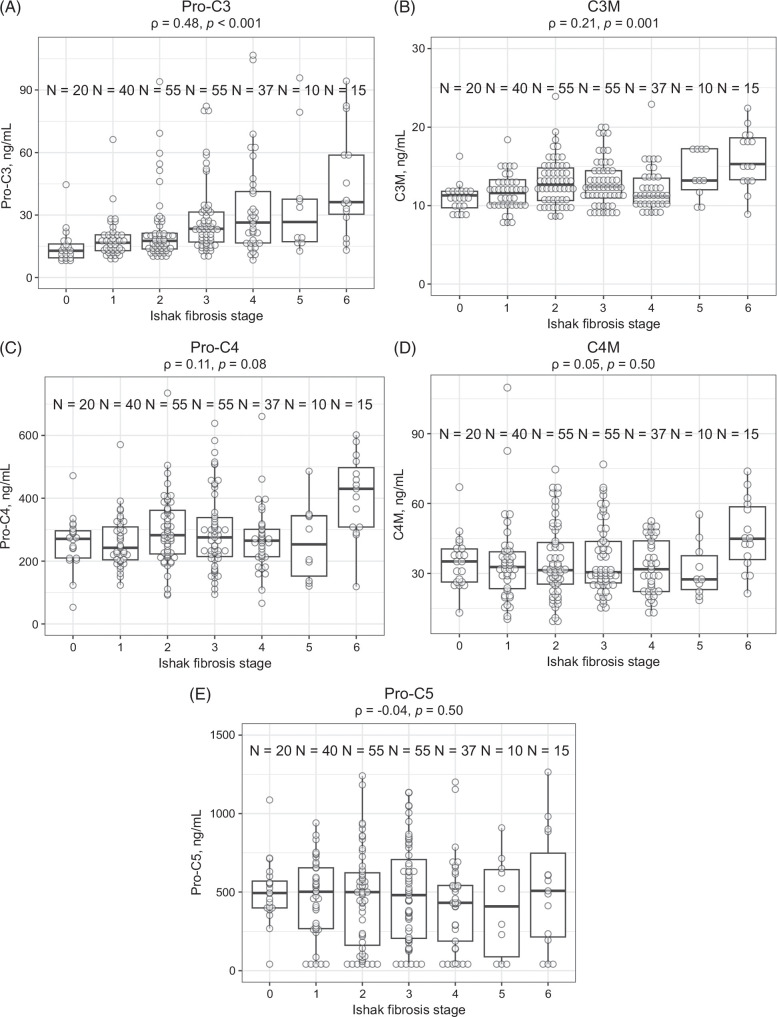
Baseline serum levels of ECM markers stratified by Ishak fibrosis stage. (A) Pro-C3, (B) C3M, (C) Pro-C4, (D) C4M, and (E) Pro-C5. Abbreviations: C3M, type III collagen matrix metalloproteinase degradation product; C4M, type IV collagen matrix metalloproteinase degradation product; ECM, extracellular matrix; Pro-C3, propeptide of type III collagen; Pro-C4, propeptide of type IV collagen; Pro-C5, propeptide of type V collagen.

At baseline, Ishak fibrosis stage was moderately correlated with the ECM marker Pro-C3 (*ρ* = 0.48; *p* < 0.001) and weakly correlated with C3M (*ρ* = 0.21; *p* = 0.001) (Figure 1A, B). Pro-C3 correlated weakly with hepatic collagen content and α-SMA (*ρ* = 0.28 and 0.27, respectively), and strongly with ELF score (*ρ* = 0.79) (all *p* < 0.001) (Supplemental Fig. S1, http://links.lww.com/HC9/A935). No correlations were observed between propeptide of type IV collagen, type IV collagen matrix metalloproteinase degradation product, or propeptide of type V collagen and Ishak fibrosis stage (Figure 1C–E).

### Diagnostic performance of ECM markers for advanced fibrosis

**FIGURE 2:**
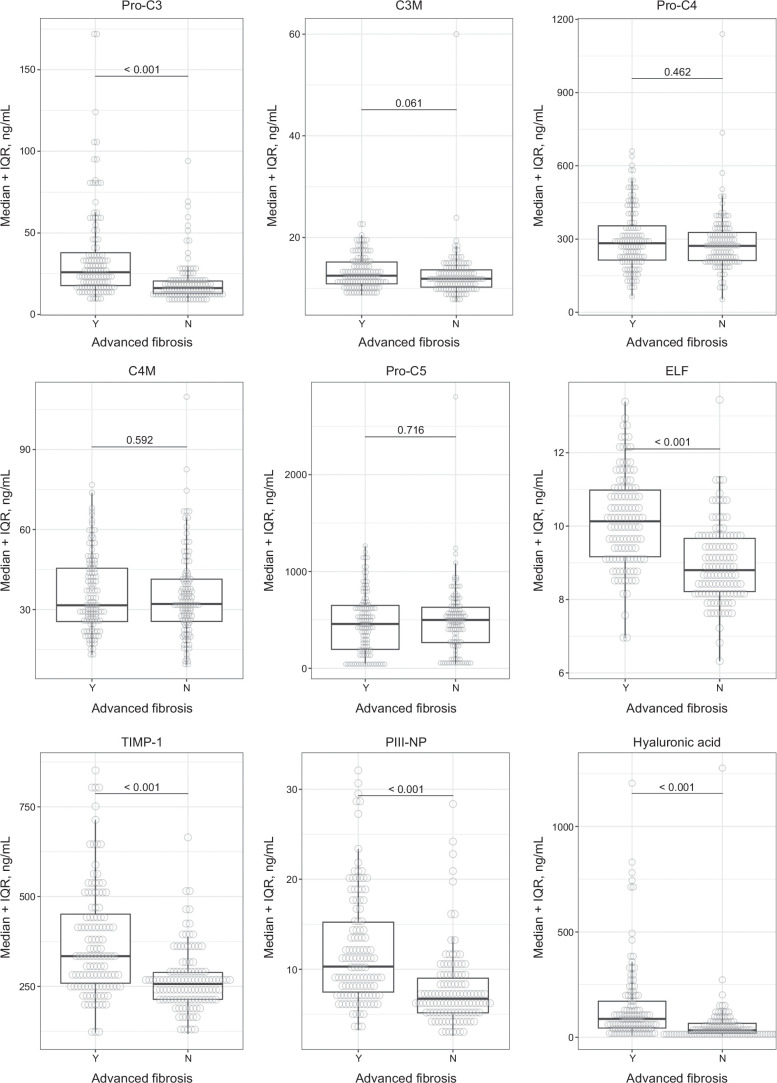
Baseline serum levels of markers of ECM remodeling in patients with and without advanced fibrosis at baseline. With advanced fibrosis (Ishak stages 3–6): n = 119; without advanced fibrosis (Ishak stages 0–2): n = 115. Abbreviations: C3M, type III collagen matrix metalloproteinase degradation product; C4M, type IV collagen matrix metalloproteinase degradation product; ECM, extracellular matrix; ELF, enhanced liver fibrosis test; PIII-NP, procollagen type III N-terminal peptide; Pro-C3, propeptide of type III collagen; Pro-C4, propeptide of type IV collagen; Pro-C5, propeptide of type V collagen; PSC, primary sclerosing cholangitis.

Median baseline Pro-C3 level and ELF score and its components (ie, TIMP-1, PIII-NP, and hyaluronic acid) were higher in patients with than without advanced fibrosis at baseline (Ishak fibrosis stages 3–6 vs. 0–2, all *p* < 0.001) (Figure 2, Supplemental Table S2, http://links.lww.com/HC9/A935). Baseline Pro-C3 and ELF score and its components provided moderate discrimination of advanced fibrosis at baseline (AUROC 0.73–0.78) (Supplemental Table S2, http://links.lww.com/HC9/A935). Optimal cutoff values of Pro-C3 and fibrosis markers for predicting advanced fibrosis are listed in Table 1. The cutoff values refer to the current study population and are exploratory. For Pro-C3, the optimal cutoff for predicting advanced fibrosis using the High Youden method was ≥ 22.2 ng/mL (sensitivity: 62%; specificity: 81%). For the ELF score, the optimal cutoff for predicting advanced fibrosis using the High Youden method was ≥ 9.86 (sensitivity: 59%; specificity: 83%). For the collagen-related ELF score component PIII-NP, the optimal cutoff for predicting advanced fibrosis using the High Youden method was ≥ 7.42 ng/mL (sensitivity: 76%; specificity: 66%). The diagnostic performance of baseline Pro-C3 was similar to that of the baseline composite biomarkers ADAPT and FIBC3 (Supplemental Fig. S2, http://links.lww.com/HC9/A935).

**TABLE 1:** Diagnostic performance of baseline Pro-C3 and fibrosis markers for advanced fibrosis (Ishak stages 3–6 vs. 0–2)

Serum marker	Method	Cutoff, ng/mL	Sensitivity, % (95% CI)	Specificity, % (95% CI)	PPV, % (95% CI)	NPV, % (95% CI)	Misclassified, % (95% CI)
Pro-C3 (n = 232)	High Youden^a^	≥ 22.2	62 (52, 70)	81 (72, 88)	77 (67, 85)	67 (59, 75)	29 (23, 35)
	High sensitivity	≥ 15.1	86 (79, 92)	45 (36, 55)	62 (54, 69)	76 (65, 86)	34 (28, 41)
	High specificity	≥ 27.1	48 (39, 57)	85 (77, 91)	77 (65, 86)	62 (54, 69)	34 (28, 40)
ELF score (n = 234)	High Youden	≥ 9.86	59 (49, 68)	83 (75, 90)	79 (69, 87)	66 (58, 74)	29 (23, 35)
	High sensitivity	≥8.77	86 (78, 91)	49 (39, 58)	63 (55, 71)	77 (65, 86)	32 (27, 39)
	High specificity	≥9.9	56 (47, 65)	85 (77, 91)	80 (70, 88)	65 (57, 73)	29 (24, 36)
TIMP-1 (n = 234)	High Youden	≥ 285.2	66 (56, 74)	74 (65, 82)	72 (63, 80)	67 (59, 76)	30 (25, 37)
	High sensitivity	≥ 234.8	86 (78, 91)	36 (27, 45)	58 (50, 65)	71 (57, 82)	39 (33, 45)
	High specificity	≥ 357	44 (35, 53)	86 (78, 92)	76 (65, 86)	60 (52, 67)	35 (29, 42)
PIII-NP (n = 234)	High Youden	≥ 7.42	76 (67, 83)	66 (57, 75)	70 (61, 78)	72 (63, 81)	29 (23, 35)
	High sensitivity	≥ 6.55	87 (79, 92)	48 (38, 57)	63 (55, 71)	77 (66, 87)	32 (27, 39)
	High specificity	≥ 10.79	49 (39, 58)	85 (77, 91)	77 (66, 86)	62 (54, 69)	33 (27, 40)
Hyaluronic acid (n = 234)	High Youden	≥ 56.58	67 (58, 76)	70 (61, 79)	70 (61, 78)	68 (58, 76)	31 (25, 38)
	High sensitivity	≥ 27.26	86 (78, 91)	43 (34, 53)	61 (53, 69)	75 (63, 84)	35 (29, 42)
	High specificity	≥ 91.76	48 (39, 57)	85 (77, 91)	77 (66, 86)	61 (53, 69)	34 (28, 40)

a Youden index=sensitivity + specificity − 1.

Abbreviations: ELF, enhanced liver fibrosis; NPV, negative predictive value; PIII-NP, procollagen type III N-terminal peptide; PPV, positive predictive value; Pro-C3, propeptide of type III collagen.

### Diagnostic performance of ECM markers for cirrhosis

**FIGURE 3:**
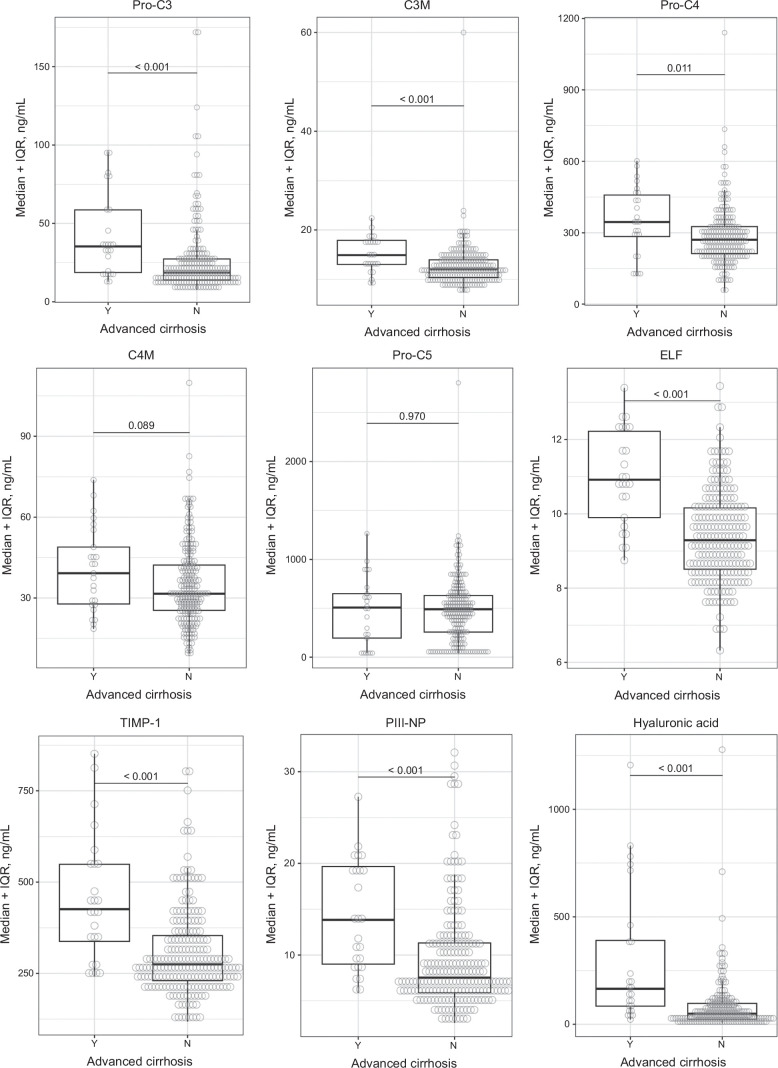
Baseline serum levels of markers of ECM remodeling in patients with and without cirrhosis at baseline. With cirrhosis (Ishak stages 5–6): n = 25; without cirrhosis (Ishak stages 0–4): n = 209. Abbreviations: C3M, type III collagen matrix metalloproteinase degradation product; C4M, type IV collagen matrix metalloproteinase degradation product; ECM, extracellular matrix; ELF, enhanced liver fibrosis test; PIII-NP, procollagen type III N-terminal peptide; Pro-C3, propeptide of type III collagen; Pro-C4, propeptide of type IV collagen; Pro-C5, propeptide of type V collagen; PSC, primary sclerosing cholangitis.

Median baseline levels of Pro-C3, propeptide of type IV collagen, C3M, ELF score, and its components were higher in patients with than without cirrhosis at baseline (Ishak fibrosis stages 5–6 vs. 0–4) (Table 2, Figure 3). Baseline Pro-C3, C3M, ELF score, and its components provided moderate discrimination of cirrhosis (AUROC 0.73–0.81) (Table 2).

**TABLE 2:** Diagnostic performance of baseline ECM and fibrosis markers for cirrhosis (Ishak stages 5–6 vs. 0–4)

		Median (Q1, Q3)			
	Serum marker	Ishak 0–4 n = 209	Ishak 5–6 n = 25	*p*	AUROC (95% CI)
ECM markers	Pro-C3, ng/mL	18.6 (13.9, 27.4)	35.3 (18.7, 58.6)	< 0.001	0.73 (0.62, 0.83)
	Pro-C4, ng/mL	270.7 (212.5, 326.2)	345.2 (283.9, 458.7)	0.011	0.66 (0.52, 0.79)
	Pro-C5, ng/mL	490.5 (252.3, 632.3)	507.5 (194.8, 649.9)	0.97	0.50 (0.37, 0.64)
	C3M, ng/mL	12 (10.3, 14)	14.9 (13.0, 17.9)	<0.001	0.73 (0.61, 0.84)
	C4M, ng/mL	31.6 (25.4, 42.4)	39.2 (27.8, 48.9)	0.09	0.60 (0.48, 0.73)
Fibrosis markers	ELF score	9.3 (8.5, 10.2)	10.9 (9.9, 12.2)	<0.001	0.81 (0.72, 0.90)
	TIMP-1, ng/mL	274.9 (230.1, 353.9)	426.2 (337.8, 549)	<0.001	0.77 (0.68, 0.87)
	PIII-NP, ng/mL	7.5 (5.9, 11.3)	13.8 (9.0, 19.7)	<0.001	0.75 (0.66, 0.84)
	Hyaluronic acid, ng/mL	49.2 (23.3, 97.2)	165 (84.2, 390.3)	<0.001	0.80 (0.71, 0.89)

Abbreviations: C3M, type III collagen matrix metalloproteinase degradation product; C4M, type IV collagen matrix metalloproteinase degradation product; ECM, extracellular matrix; ELF, enhanced liver fibrosis; PIII-NP, procollagen type III N-terminal peptide; Pro-C3, propeptide of type III collagen; Pro-C4, propeptide of type IV collagen; Pro-C5, propeptide of type V collagen; Q, quartile.

### Prognostic performance of ECM markers for PSC-related clinical events

During a median follow-up of 23.0 months (range: 3.7–27.8), 47 of 234 patients (20%) experienced at least 1 PSC-related clinical event, with ascending cholangitis being the most common first event (n = 27). Patients with higher levels of Pro-C3, type IV collagen matrix metalloproteinase degradation product, alkaline phosphatase, ELF score, PIII-NP, Mayo risk, and FibroTest scores, and those at a higher Ishak fibrosis stage at baseline were more likely than those with lower levels to develop PSC-related clinical events (all HRs and the lower limit of 95% CIs > 1) (Table 3). Baseline Pro-C3, ELF score, PIII-NP, Mayo risk score, alkaline phosphatase, FibroTest score, and Ishak fibrosis stage showed close to moderate prognostic ability for PSC-related clinical events (AUROC 0.69–0.72) (Table 3). The prognostic performances of the noninvasive markers Pro-C3 (AUROC 0.70) and ELF score (AUROC 0.71) were similar to that of Ishak fibrosis stage (AUROC 0.69) (Table 3, Supplemental Fig. S3, http://links.lww.com/HC9/A935).

**TABLE 3:** Prognostic performance of baseline ECM markers, other noninvasive markers, and histology for PSC-related clinical events

		Median (Q1, Q3)			
	Serum marker	Clinical events (n = 47)^a^	No clinical events (n = 187)	HR (95% CI)	AUROC (95% CI)
ECM markers	Pro-C3, ng/mL	27.1 (18.8, 41.6)	17.9 (13.5, 27.2)	1.015 (1.007, 1.022)	0.70 (0.62, 0.78)
	Pro-C4, ng/mL	298.6 (228.4, 363.4)	271.6 (210.7, 335.3)	1.0008 (0.9989, 1.0028)	0.57 (0.47, 0.66)
	Pro-C5, ng/mL	533.7 (337.6, 692.6)	458.6 (230.2, 630.5)	1.0004 (0.9997, 1.0011)	0.57 (0.47, 0.66)
	C3M, ng/mL	13.4 (11.4, 17)	11.9 (10.3, 13.7)	1.034 (0.997, 1.072)	0.66 (0.57, 0.75)
	C4M, ng/mL	37.4 (28.8, 49.9)	31.4 (24.6, 41.7)	1.018 (1.002, 1.034)	0.62 (0.54, 0.71)
Other markers	ELF score	10.3 (9.7, 10.9)	9.2 (8.5, 10.1)	1.534 (1.261, 1.867)	0.71 (0.63, 0.79)
	PIII-NP, ng/mL	11.2 (8.7, 17.1)	7.3 (5.9, 11)	1.08 (1.043, 1.126)	0.70 (0.61, 0.78)
	TIMP-1, ng/mL	407 (279.2, 474.8)	268.3 (230.1, 348.1)	1.004 (1.002, 1.005)	0.71 (0.62, 0.80)
	Hyaluronic acid, ng/mL	92.9 (51.6, 157.2)	46.1 (22.4, 97.9)	1.001 (1.000, 1.002)	0.68 (0.60, 0.76)
	Mayo risk score	0.4 (0, 1)	−0.2 (−0.7, 0.3)	2.04 (1.54, 2.70)	0.72 (0.65, 0.80)
	ALP, U/L	381 (271, 622)	226 (122, 345)	1.0017 (1.0009, 1.0024)	0.71 (0.62, 0.79)
	FibroTest	0.57 (0.41, 0.71)	0.37 (0.22, 0.53)	12.23 (3.63, 41.18)	0.69 (0.61, 0.77)
Histology	Ishak fibrosis stage	3 (2, 4)	2 (1, 3)	1.45 (1.22, 1.72)	0.69 (0.61, 0.77)

*Note*: Data are presented as median (Q1, Q3).

a First clinical events were ascending cholangitis (n = 27), jaundice (n = 10), cholangiocarcinoma (n = 3), ascites (n = 2), HE (n = 2), variceal hemorrhage (n = 2), and sepsis (n = 1).

Abbreviations: ALP, alkaline phosphatase; C3M, type III collagen matrix metalloproteinase degradation product; C4M, type IV collagen matrix metalloproteinase degradation product; ECM, extracellular matrix; ELF, enhanced liver fibrosis; PIII-NP, procollagen type III N-terminal peptide; Pro-C3, propeptide of type III collagen; Pro-C4, propeptide of type IV collagen; Pro-C5, propeptide of type V collagen; PSC, primary sclerosing cholangitis; Q, quartile.

### Prognostic performance of ECM markers for fibrosis progression

**FIGURE 4:**
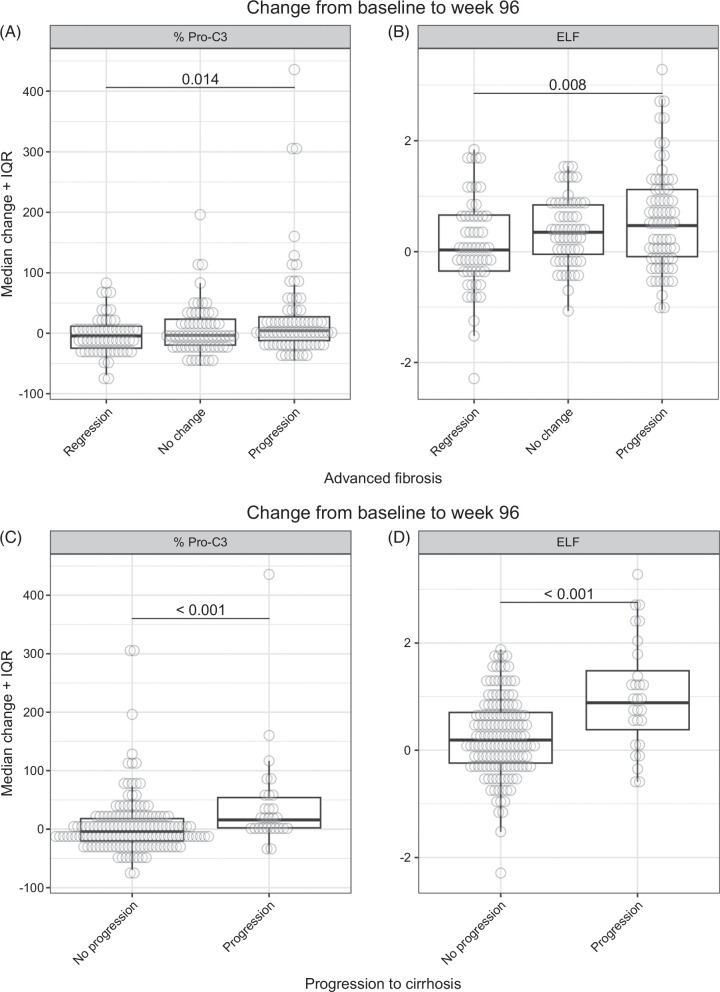
Change from baseline to week 96 in Pro-C3 levels and ELF score according to change in fibrosis stage or progression to cirrhosis at week 96. (A) Pro-C3 and (B) ELF in patients with fibrosis regression (n = 62), no change in fibrosis (n = 74), and fibrosis progression (n = 80). (C) Pro-C3 and (D) ELF in patients with cirrhosis progression (n=30) and without progression to cirrhosis (n=161). Missing data at week 96 were imputed using available data at week 48. Abbreviations: ELF, enhanced liver fibrosis; Pro-C3, propeptide of type III collagen.

In total, 216 patients had Ishak scores at both baseline and week 48 or week 96. Of these, 27 had missing data at week 96 that were imputed using available week 48 data. Overall, 62 (28.7%) of these 216 patients experienced regression of fibrosis, 74 (34.3%) had no change in fibrosis, and 80 (37.0%) had fibrosis progression at week 96. A trend of increased median in Pro-C3 and ELF from baseline to week 96 was observed with fibrosis progression (regressed, unchanged, or progressed, in that order) at week 96 (*p* for trend: 0.014 and 0.008, respectively) (Figure 4A, B). In contrast, no increasing or decreasing trend was observed for median changes from baseline to week 48 (Supplemental Fig. S4, http://links.lww.com/HC9/A935).

Of 191 patients without cirrhosis at baseline and having biopsy data at week 48 or week 96 (in which missing biopsy data at week 96 were imputed using available week 48 data), 30 (15.7%) had progressed to cirrhosis at week 96. Median changes in Pro-C3 and ELF from baseline to week 96 were higher in patients who progressed to cirrhosis than in those without progression to cirrhosis (both *p* < 0.001) (Figure 4C, D).

### Intraindividual variability in Pro-C3, ELF, and ELF components

Intraindividual coefficients of variation for Pro-C3, ELF score, PIII-NP, TIMP-1, and hyaluronic acid were 17.0%, 4.8%, 18.6%, 16.4%, and 40.4%, based on data collected at baseline, week 12, and week 24. Coefficients of variation were similar across fibrosis stages for each of the markers (Supplemental Table S3, http://links.lww.com/HC9/A935).

## DISCUSSION

Results from this 96-week phase 2b study of simtuzumab in patients with PSC show the potential utility of markers of ECM remodeling for diagnostic and prognostic purposes in PSC. The collagen formation marker Pro-C3 was shown to correlate with the fibrosis stage, and Pro-C3 and ELF had utility for identifying advanced fibrosis and cirrhosis and predicting PSC-related events and fibrosis progression.

ECM remodeling is dysregulated in liver fibrosis. We hypothesized that ECM remodeling would result in changes in serum levels of collagen formation and degradation markers. In the present study, serum levels of the collagen formation marker Pro-C3 rose with increasing Ishak fibrosis stage, suggesting increased ECM remodeling in advanced disease. Accordingly, Pro-C3 provided discrimination of advanced fibrosis with moderate strength. Baseline Pro-C3 serum levels had moderate diagnostic and prognostic ability, similar to ELF and its components. Of the other ECM markers, baseline C3M serum levels had moderate diagnostic ability but only weak prognostic value.

Pro-C3 serum levels and ELF score were reduced from baseline at week 12 (exploratory endpoints) in the phase 2 study of aldafermin in patients with PSC.^4^ Levels of circulating bile acids were reduced with aldafermin and correlated with changes in Pro-C3 and ELF score.^5^ The observation in the current analysis that patients with higher levels of Pro-C3 had an increased likelihood of developing PSC-related clinical events is supported by a study on transplant-free survival in large-duct PSC.^15^ In that study, patients with high baseline Pro-C3 levels had a significantly shorter transplant-free survival time than patients with low baseline marker levels.^15^ Higher baseline Pro-C3 levels were associated with greater fibrosis progression in a study in patients with chronic hepatis C.^16^ In the study in large-duct PSC, patients with high ELF scores at baseline had significantly shorter transplant-free survival times than patients with low baseline ELF scores.^15^

Histology is the gold standard for prognosis. However, disease activity distribution is patchy in PSC, and biopsies are limited by sampling variability. There are no approved surrogate end point markers for patients with PSC in clinical trials, and markers with prognostic value for PSC-related clinical events are therefore needed. The diagnostic performance of Pro-C3 for advanced fibrosis in patients with PSC was at least similar to that of the Pro-C3-derived composite biomarkers ADAPT and FIBC3, which were originally developed for fibrosis detection in patients with metabolic dysfunction-associated steatotic liver disease.^13^,^14^ The ELF score was shown previously in this study population to correlate with the fibrosis stage (*ρ* = 0.55; *p* < 0.001).^2^ In addition, both baseline ELF score and change in score over 12 weeks were significantly associated with the occurrence of clinical events.^2^ The optimal threshold for predicting clinical events was a change in ELF score of 0.19 over 12 weeks.^2^ Pro-C3 is a single biomarker for type III collagen homeostasis. The biological variation of Pro-C3 was about 20% in the current study, which speaks to its dynamics. Pro-C3, ELF score, FibroTest, Mayo risk score, and alkaline phosphatase showed similar prognostic performances for PSC-related events to Ishak fibrosis stage, suggesting that these noninvasive measures could provide an alternative option to liver biopsy as prognostic tools. Intraindividual variation of Pro-C3, ELF, and ELF components were similar in PSC with mild and advanced fibrosis stages. The lower intraindividual coefficient obtained for ELF (4.8%) is likely to be mainly due to the ELF score being calculated using log transformation.

PIII-NP and Pro-C3 are both biomarkers derived from type III collagen turnover.^7^ Both form part of the N-terminal propeptide. The Pro-C3 antibody targets the exact epitope exposed owing to ADAM-TS2-mediated release of the type III collagen propeptide fragment.^7^ The exact PIII-NP assay epitope within the N-terminal propeptide is unknown.^7^ In the current study, PIII-NP and Pro-C3 showed very similar diagnostic performance and prognostic performance, which is likely due to homeostasis of collagen formation and degradation.

The design of this prospective phase 2b trial was exceptional in that it included a biopsy requirement, which provided a unique opportunity to evaluate serum ECM markers in relation to histology. The study has important strengths, including the prospective data collection, rigorous methodology, and the large, well-defined population of patients with PSC. Fibrosis staging was based on liver biopsy. The number of clinical events and patients with fibrosis progression enabled the assessment of the prognostic performance of ECM markers for PSC. Study limitations include the absence of a validation cohort, which means that the identified diagnostic cutoff values are exploratory only. Liver stiffness measurements were only available for a subset (n = 58) of the study population,^2^ and the measure was thus not included in the current analysis.

In conclusion, in this phase 2b study, the collagen formation marker Pro-C3 correlated with fibrosis stage, and Pro-C3 and ELF identified advanced fibrosis and cirrhosis and predicted PSC-related events and fibrosis progression. The results support the clinical utility of Pro-C3 and ELF as diagnostic and prognostic markers in PSC and their ongoing evaluation as endpoints within clinical trials in PSC.

## Supplementary Material

SUPPLEMENTARY MATERIAL
