# Supplementary material for: Serologic extracellular matrix remodeling markers are related to fibrosis stage and prognosis in a phase 2b trial of simtuzumab in patients with primary sclerosing cholangitis
Source: Hepatol Commun. 2024 Jul 5;8(7):e0467. doi: 10.1097/HC9.0000000000000467 (PMC11227354; doi:10.1097/HC9.0000000000000467)
Supplement: SUPPLEMENTARY MATERIAL [file hc9-8-e0467-s001.docx]

Serologic extracellular matrix remodeling markers are related to fibrosis stage and prognosis in primary sclerosing cholangitis

Douglas Thorburn, Diana J. Leeming, William Barchuck, Ya Wang, Xiaomin Lu, Vladislav A. Malkov, Kaori L. Ito, Christopher L. Bowlus, Cynthia Levy, Zachary Goodman, Morten A. Karsdal, Andrew J. Muir, Jun Xu

Table of contents

Supplementary tables ……………………………………………………………………2

Supplementary figures ...………………………………………...…………….….6

## Supplementary tables

**Table S1.** **Baseline demographics and clinical characteristics.**

| **Characteristics** | **All patients (N = 234)** |
| --- | --- |
| Demographics |  |
| Age, years, median (Q1, Q3) | 45 (37, 52) |
| Men, n (%) | 149 (64) |
| Ulcerative colitis, n (%) | 112 (48) |
| UDCA therapy, n (%) | 144 (62) |
| Liver biochemistry and prognostic indices, median (Q1, Q3) | |
| ALP, U/L | 260 (129, 401) |
| ALT, U/L | 62 (35, 112) |
| AST, U/L | 51 (33, 83) |
| GGT, U/L | 236 (99, 524) |
| Platelets, × 10^3^/µL | 255 (197, 312) |
| Direct Bilirubin, mg/dL | 0.2 (0.2, 0.4) |
| Albumin, g/dL | 4.1 (3.7, 4.3) |
| Mayo risk score | −0.12 (−0.58, 0.42) |
| MELD score | 7 (6, 8) |
| Serum markers of bile acid homeostasis, median (Q1, Q3) | |
| C4, ng/mL | 11.5 (4.7, 21.4) |
| Primary bile acid, ng/mL | 1965 (980, 7017) |
| Total bile acid, ng/mL | 5409 (2,314, 14,135) |
| Serum markers of ECM remodeling, median (Q1, Q3) | |
| Pro-C3, ng/mL | 19.8 (14.2, 30.5) |
| Pro-C4, ng/mL | 275.6 (212.6, 342.0) |
| Pro-C5, ng/mL | 491.3 (236.3, 636.9) |
| C3M, ng/mL | 12.2 (10.5, 14.5) |
| C4M, ng/mL | 31.9 (25.5, 43.5) |
| Serum markers of fibrosis, median (Q1, Q3) | |
| ELF score | 9.5 (8.6, 10.4) |
| TIMP-1, ng/mL | 278.8 (235.0, 383.0) |
| PIII-NP, ng/mL | 8.3 (6.0, 11.8) |
| Hyaluronic acid, ng/mL | 54.9 (24.0, 107.9) |
| FibroTest/FibroSure | 0.41 (0.23, 0.60) |
| Liver histology |  |
| Ishak fibrosis stage, n (%) |  |
| 0–2 | 115 (49) |
| 3–4 | 94 (40) |
| 5–6 | 25 (11) |
| Hepatic collagen content, %, median (Q1, Q3) | 4.4 (2.7, 7.0) |
| α-SMA expression, %, median (Q1, Q3) | 2.5 (1.3, 5.6) |

α-SMA, α-smooth muscle actin; ALP, alkaline phosphatase; ALT, alanine aminotransferase; AST, aspartate aminotransferase; C3M, type III collagen matrix metalloproteinase degradation product; C4, 7α-hydroxy-4-cholesten-3-one; C4M, type IV collagen matrix metalloproteinase degradation product; ECM, extracellular matrix; ELF, enhanced liver fibrosis; GGT, γ-glutamyl transferase; MELD, model for end-stage liver disease; PIII-NP, procollagen type III N-terminal peptide; Pro-C3, propeptide of type III collagen; Pro-C4, propeptide of type IV collagen; Pro-C5, propeptide of type V collagen; Q, quartile; TIMP-1, tissue inhibitor of metalloproteinase-1; UDCA, ursodeoxycholic acid.

**Table S2.** **Diagnostic performance of baseline ECM and fibrosis markers for advanced fibrosis (Ishak stages 3–6 vs 0–2).**

|  | **Serum Marker** | **Ishak 0–2**  **n = 115** | **Ishak 3–6**  **n = 119** | ***p* value** | **AUROC (95% CI)** |
| --- | --- | --- | --- | --- | --- |
|  |  | **Median (Q1, Q3)** | |  |  |
| ECM markers | Pro-C3, ng/mL | 16.2 (12.7, 20.6) | 25.9 (17.7, 37.9) | <0.001 | 0.75 (0.69, 0.81) |
|  | Pro-C4, ng/mL | 272.4 (212.3, 328.6) | 283.2 (214.2, 354.6) | 0.46 | 0.53 (0.45, 0.60) |
|  | Pro-C5, ng/mL | 499.6 (263.6, 630.7) | 458 (195.7, 649.9) | 0.72 | 0.51^a^ (0.44, 0.59) |
|  | C3M, ng/mL | 11.9 (10.2, 13.7) | 12.5 (10.9, 15.2) | 0.06 | 0.57 (0.50, 0.64) |
|  | C4M, ng/mL | 32.1 (25.2, 41.6) | 31.6 (25.5, 45.5) | 0.59 | 0.52 (0.45, 0.60) |
| Fibrosis markers | ELF score | 8.8 (8.2, 9.7) | 10.1 (9.2, 11) | <0.001 | 0.78 (0.72, 0.83) |
|  | TIMP-1, ng/mL | 256.8 (212.4, 289.8) | 334.2 (257.9, 452.7) | <0.001 | 0.73 (0.66, 0.79) |
|  | PIII-NP, ng/mL | 6.7 (5.1, 9) | 10.3 (7.4, 15.3) | <0.001 | 0.75 (0.69, 0.82) |
|  | Hyaluronic acid, ng/mL | 33.1 (19, 67.3) | 87 (43.5, 177.3) | <0.001 | 0.75 (0.69, 0.81) |

^a^Negative association between the marker and advanced fibrosis.

Data are presented as median (Q1, Q3).

AUROC, area under the receiver operating characteristic curve; C3M, type III collagen matrix metalloproteinase degradation product; C4M, type IV collagen matrix metalloproteinase degradation product; CI, confidence interval; ECM, extracellular matrix; ELF, enhanced liver fibrosis; PIII-NP, procollagen type III N-terminal peptide; Pro-C3, propeptide of type III collagen; Pro-C4, propeptide of type IV collagen; Pro-C5, propeptide of type V collagen; Q, quartile; TIMP-1, tissue inhibitor of metalloproteinase-1.

**Table S3. Intra-patient variability in serum markers of ECM remodeling and fibrosis.**

| **Serum markers** | **Ishak fibrosis stage** | **n** | **Intra-patient CV, %** |
| --- | --- | --- | --- |
| Pro-C3 | 0–2 | 107 | 15.05 |
|  | 3–4 | 75 | 19.45 |
|  | 5–6 | 23 | 16.71 |
| ELF score | 0–2 | 106 | 5.20 |
|  | 3–4 | 75 | 4.32 |
|  | 5–6 | 23 | 4.10 |
| TIMP-1 | 0–2 | 106 | 16.12 |
|  | 3–4 | 75 | 17.05 |
|  | 5–6 | 23 | 15.77 |
| PIII-NP | 0–2 | 106 | 19.74 |
|  | 3–4 | 75 | 17.13 |
|  | 5–6 | 23 | 17.90 |
| Hyaluronic acid | 0–2 | 106 | 42.90 |
|  | 3–4 | 75 | 38.49 |
|  | 5–6 | 23 | 34.73 |

CV, coefficient of variation; ECM, extracellular matrix; ELF, enhanced liver fibrosis; PIII-NP, procollagen type III N-terminal peptide; Pro-C3, propeptide of type III collagen; TIMP-1, tissue inhibitor of metalloproteinase-1.

## Supplementary figures


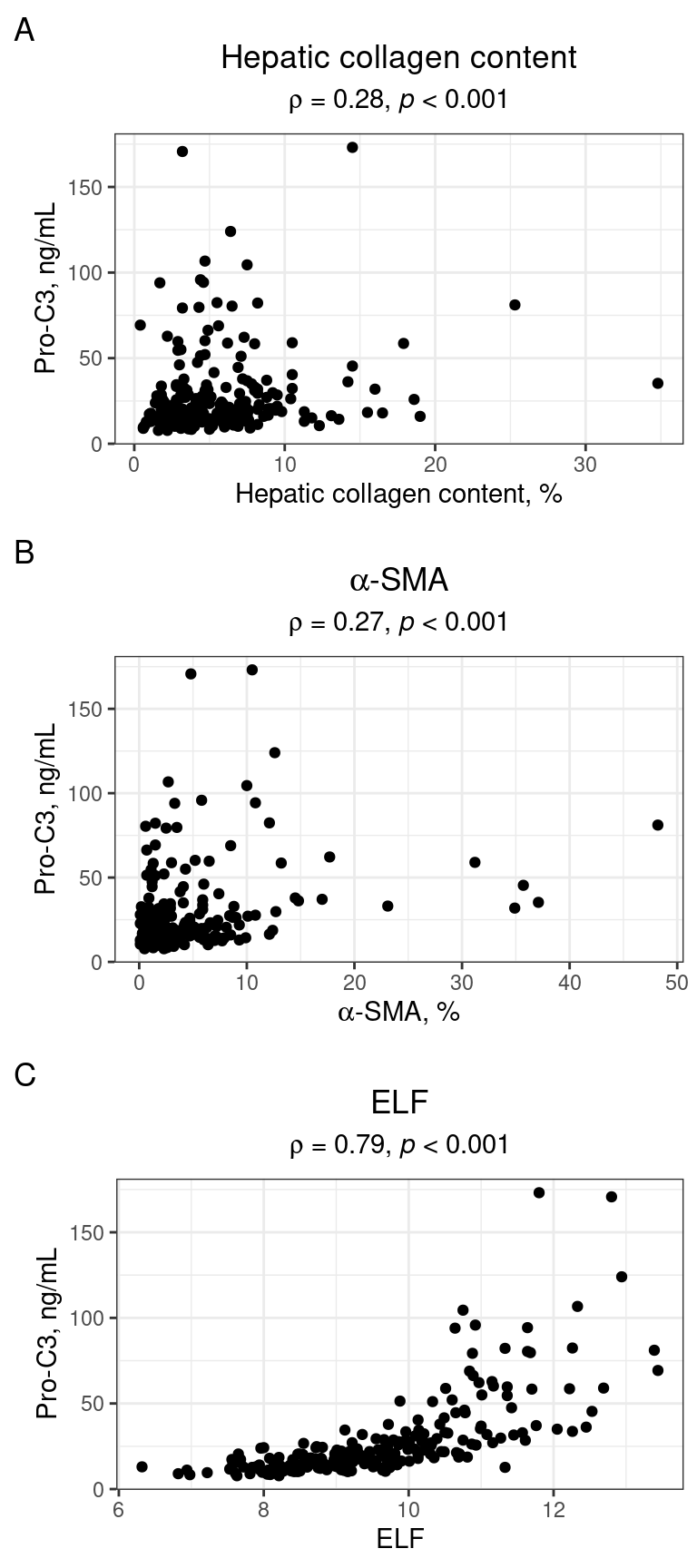


**Fig. S1.** **Baseline serum levels of Pro-C3 plotted against liver fibrosis markers.** (A) Hepatic collagen content, (B) α-SMA, (C) ELF score.

α-SMA, α-smooth muscle actin; ELF, enhanced liver fibrosis; Pro-C3, propeptide of type III collagen.


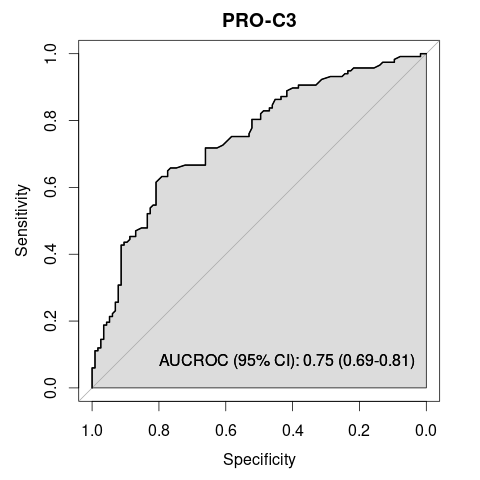


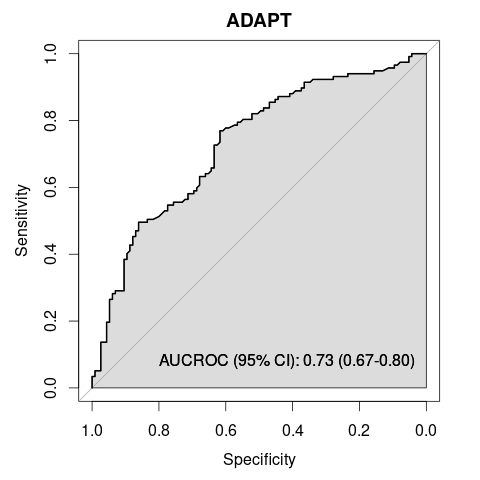


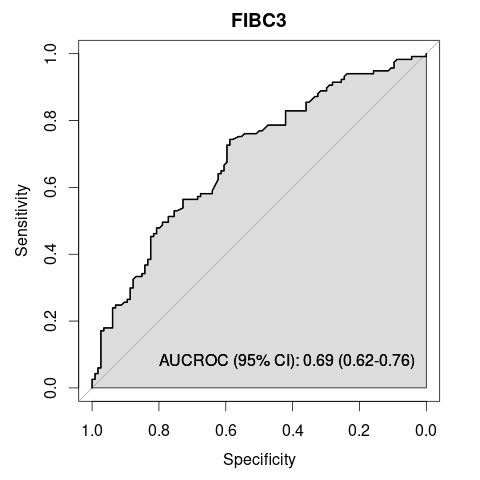


**Fig. S2.** **ROC curves for sensitivity and specificity of baseline Pro-C3 and the composite biomarkers ADAPT and FIBC3 for detecting patients with advanced fibrosis (Ishak stages 3–6 vs 0–2).**
ADAPT and FIBC3 scores were calculated using published algorithms:^13,14^

$A\mathrm{DAPT}=\exp(\log10 (\frac{(\mathrm{age}\times\mathrm{Pro}-C3)}{\surd\mathrm{platelets}}))+T2\mathrm{DM}$

FIBC3 = −5.939 + (0.053 × age) + (0.076 × BMI) + (1.614 × T2DM) – (0.009 × platelets) + (0.071 × Pro-C3)

ADAPT, age, presence of diabetes, Pro-C3, and platelet count; AUROC, area under the receiver operating characteristic curve; BMI, body mass index; CI, confidence interval; Pro-C3, propeptide of type III collagen; ROC, receiver operating characteristic; T2DM, type 2 diabetes mellitus.


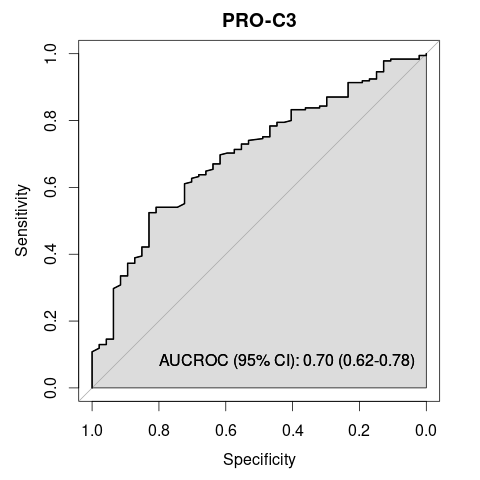


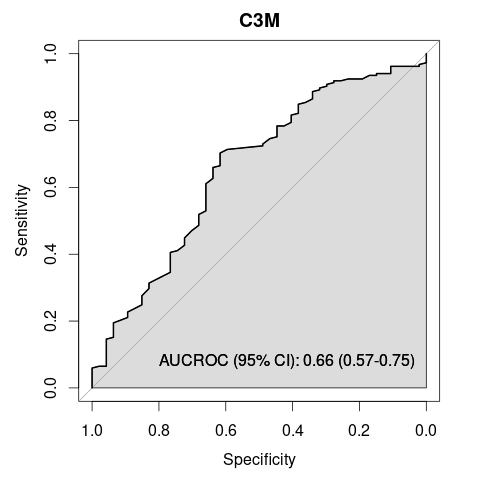


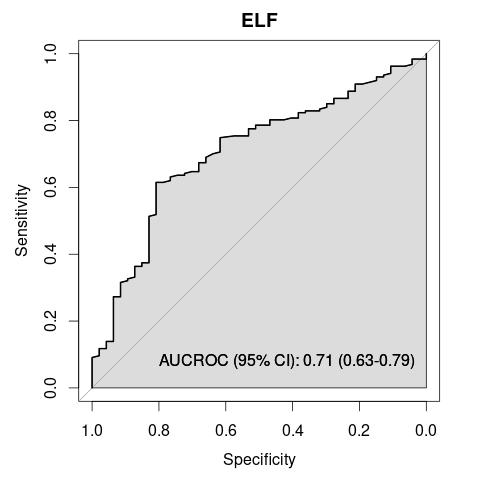


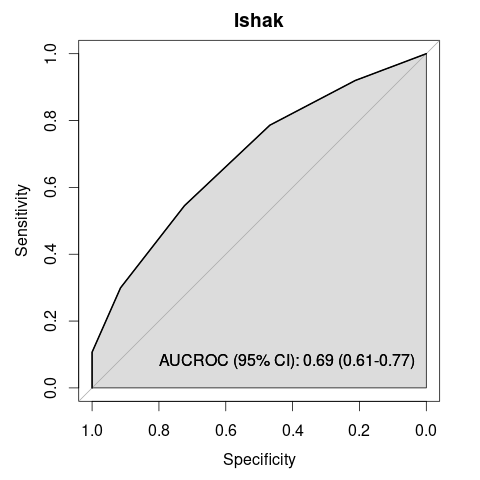


**Fig. S3.** **Prognostic performance of baseline markers of ECM remodeling for PSC-related clinical events.**

AUROC, area under the receiver operating characteristic curve; C3M, type III collagen matrix metalloproteinase degradation product; CI, confidence interval; ECM, extracellular matrix; ELF, enhanced liver fibrosis; Pro-C3, propeptide of type III collagen; PSC, primary sclerosing cholangitis.


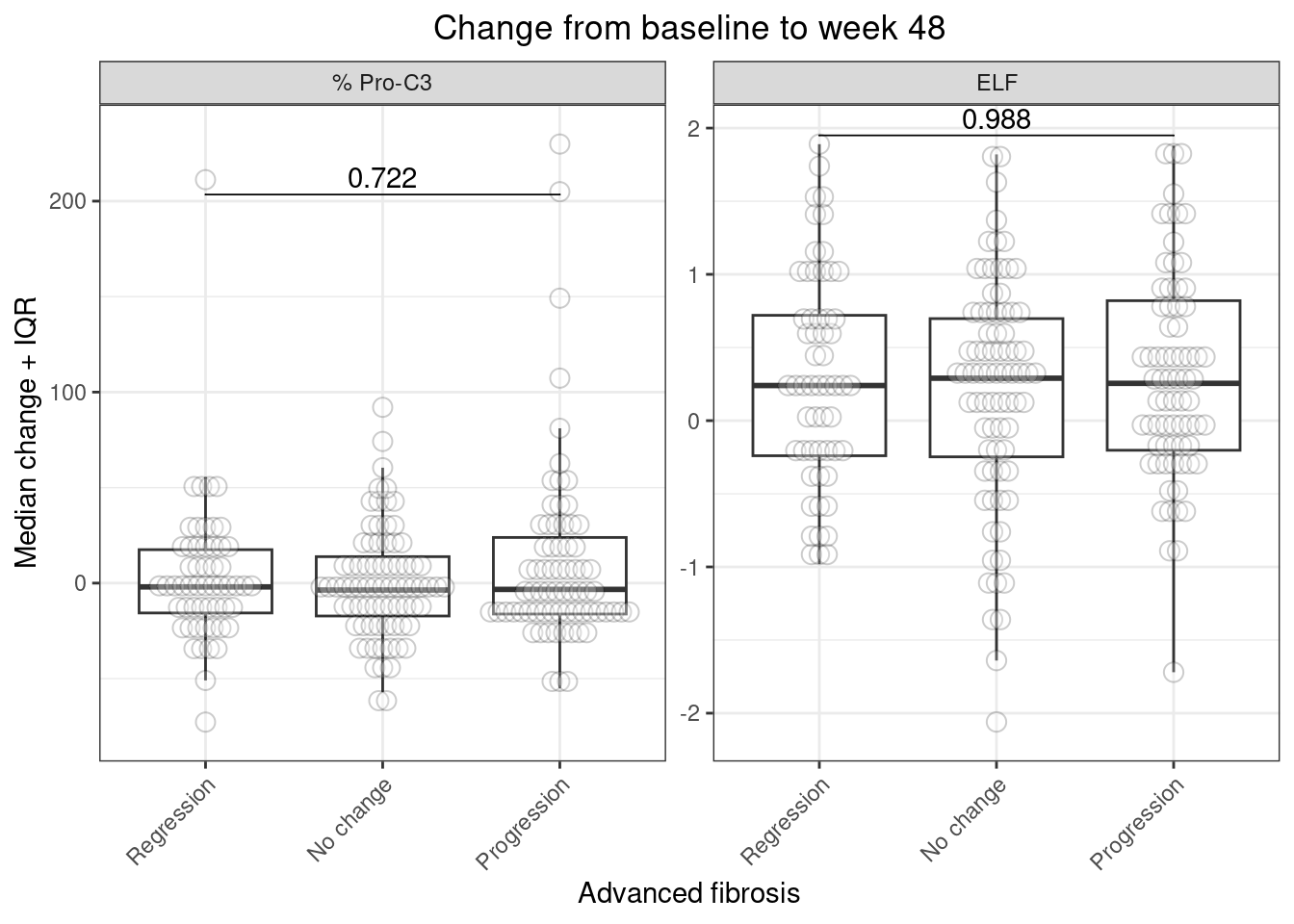


**Fig. S4.** **Change from baseline to week 48 in Pro-C3 levels and ELF score according to change in fibrosis stage at week 48.** (A) % Change in Pro-C3 and (B) ELF in patients with fibrosis regression (n = 58), no change in fibrosis (n = 80), and fibrosis progression (n = 74).

ELF, enhanced liver fibrosis; IQR, interquartile range; Pro-C3, propeptide of type III collagen; PSC, primary sclerosing cholangitis.
